# Foveal Microvascular Structures in Eyes with Silicone Oil Tamponade for Rhegmatogenous Retinal Detachment: A Swept-source Optical Coherence Tomography Angiography Study

**DOI:** 10.1038/s41598-020-59504-3

**Published:** 2020-02-13

**Authors:** Jong Young Lee, Jin Young Kim, Sang-Yoon Lee, Jin Ho Jeong, Eun Kyoung Lee

**Affiliations:** 1 Department of Ophthalmology, Jeju National University School of Medicine, Jeju National University Hospital, Jeju, Korea; 2 Department of Ophthalmology, Seoul National University College of Medicine, Seoul National University Hospital, Seoul, Korea

**Keywords:** Diseases, Medical research, Pathogenesis

## Abstract

Silicone oil (SO) is widely used as a long-term intravitreal tamponading agent for rhegmatogenous retinal detachment (RRD) repair. This study investigated the structural changes of the foveal microvasculature using optical coherence tomography angiography (OCTA) in patients with RRD treated with vitrectomy and SO tamponade. Thirty-eight patients with unilateral RRD who were treated with vitrectomy and SO tamponade and were followed up for ≥3 months after SO removal were included. *En face* OCTA images were obtained and foveal avascular zone (FAZ) area and vascular density (VD) were compared between study eyes and unaffected contralateral eyes. The FAZ area in deep capillary plexus (DCP) was larger (*P* < 0.001) and the VD in DCP was lower (*P* = 0.022) in the study eyes than in the fellow eyes. The duration of SO tamponade was significantly correlated with the enlargement of FAZ area (*P* = 0.034) and reduction of VD in DCP (*P* = 0.015). These changes could reflect vascular insufficiency in eyes with SO tamponade and may represent a potential explanation for the pathogenesis of retinal thinning and unexplained visual loss.

## Introduction

Silicone oil is widely used as a long-term intravitreal tamponading agent in vitreoretinal surgery for the treatment of complex retinal detachments^1^. For repairing rhegmatogenous retinal detachment (RRD), silicone oil provides excellent structural support to maintain retinal attachment due to its high viscosity and surface tension. However, the use of silicone oil has been associated with complications including cataract^2^, intraocular pressure (IOP) rise^3^, emulsification of oil with secondary glaucoma^4^, subretinal migration of oil^5^, and band keratopathy^6^. Furthermore, unexplained loss of vision has occasionally been reported in association with the use of silicone oil^7–9^. Previous studies have reported that visual loss after silicone oil use was associated with a significant reduction in inner retinal thickness in the macular area^10^. A more recent study by Lee *et al*.^11^ showed that reduction in thickness is not restricted to the inner retinal layer but also in the outer retinal layer as well. Furthermore, a reduction of the ganglion cell layer, outer plexiform layer, and outer nuclear layer thicknesses is correlated to worse visual outcome. Despite these knowledges, the precise pathogenesis of this complication remains obscure.

Optical coherence tomography angiography (OCTA) is a noninvasive imaging modality that provides depth-resolved imaging of retinal vasculature. The brief principle of OCTA involves determining the changes in consecutive B-scans at the same location and comparing the decorrelation signal intensity or amplitude between them. To be precise, the B-scan detects the flow of erythrocytes through retinal blood vessels. The decorrelation analysis by software generates the ultimate result with OCTA image within few seconds. The OCTA improved the visualization of the chorioretinal microvasculature without dye injection and allowed layer-by-layer analysis of the different retinal vascular plexuses. Although there have been several studies of eyes with silicone oil tamponade using spectral-domain optical coherence tomography (SD-OCT)^11–13^, changes in foveal microvascular structures in response to silicone oil using OCTA have not yet been investigated.

The aim of this study was to evaluate foveal microvascular structures of patients who underwent silicone oil injection for the treatment of RRD. We compared changes in retinal thickness and foveal microvasculature between the treated and the normal contralateral eyes 3 months after silicone oil removal. Furthermore, factors related to changes in foveal microvasculature were also evaluated.

## Results

Of the 56 eyes enrolled initially, 18 eyes were dropped out because a loss of follow-up in 7 eyes, secondary epiretinal membrane (ERM) in 5 eyes, proliferative vitreoretinopathy (PVR) grade greater than C in 3 eyes, macular edema in 2 eyes, and retinal re-detachment in 1 eye. The remaining 38 eyes included in this study were imaged using both SD-OCT and OCTA. Patient demographics and baseline characteristics are shown in Table 1. The mean age of patients at the time of presentation was 57.68 ± 12.68 years and the mean duration of RRD before surgery was 9.23 ± 12.27 days. At the time of surgery, 27 eyes (71.1%) showed macula-off RRD and 8 eyes (21.1%) demonstrated PVR. Fourteen eyes (36.8%) experienced increased IOP during the early postoperative period. Topical anti-glaucoma medication was sufficient to control IOP in those eyes and no eyes required glaucoma surgery. The mean duration of silicone oil tamponade was 4.46 ± 1.19 months and the mean follow up periods was 7.34 ± 3.68 months.

**Table 1:** Demographics and baseline characteristics of the study participants.

Variable	Study eyes
Patients	38
Age at presentation (years)	57.68 ± 12.68
Male/Female (%)	24/14 (63.2/36.8)
Spherical equivalent (diopters)	−1.73 ± 2.48
Axial length (mm)	24.14 ± 1.43
Diabetes (%)	7 (18.4)
Hypertension (%)	10 (26.3)
Duration of RRD before surgery (days)	9.23 ± 12.27
Preoperative factors (RRD)	
Macula on/off (%)	11/27 (28.9/71.1)
Number of quadrants involved	2.87 ± 0.60
PVR (%), Grade A/B	8 (21.1), 6/2
Phakic/Pseudophakic (%)	20/18 (52.6/47.4)
Intraoperative factors	
Combined cataract surgery (%)	19 (50)
Perfluorocarbon liquid use (%)	34 (89.5)
Operation time (min)	126.94 ± 39.22
Volume of SO injected (ml)	6.34 ± 1.06
Postoperative factors	
IIOP during SO tamponade (%)	14 (36.8)
Duration of SO tamponade (months)	4.46 ± 1.19
Follow up periods (months)	7.34 ± 3.68

RRD = rhegmatogenous retinal detachment; PVR = proliferative vitreoretinopathy; SO = silicone oil; IIOP = increased intraocular pressure.

Values are presented as mean ± SD unless otherwise indicated.

**Figure 1:**
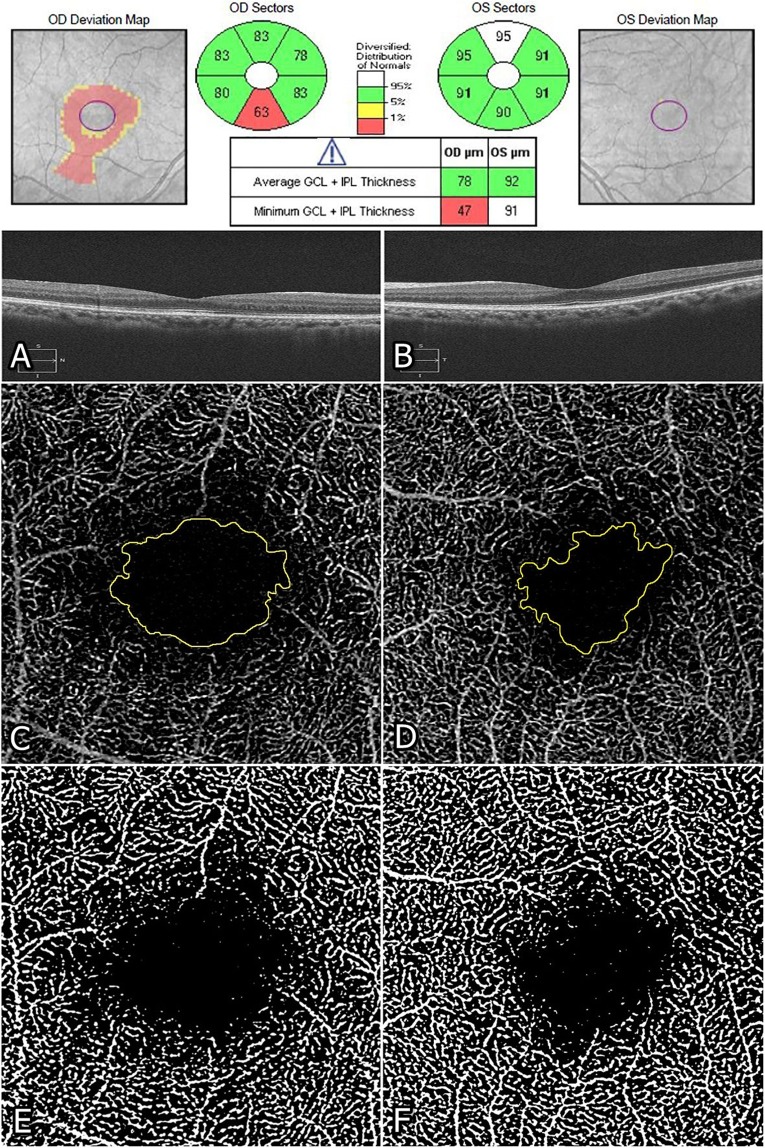
Representative cases of rhegmatogenous retinal detachment in the right eye operated with silicone oil and the normal contralateral left eye. Optical coherence tomography (OCT) (**A, B**), optical coherence tomography angiography (OCTA) (**C, D**), and converted binary images (**E, F**) taken 3 months after silicone oil removal surgery in a 45-year-old woman. (**A, B**) Central foveal thickness (CFT) and macular ganglion cell-inner plexiform layer (mGCIPL) thickness were lower in the study eye (234 μm and 78 μm, respectively) than in the contralateral eye (262 μm and 92 μm, respectively). (**C, D**) Foveal avascular zone (FAZ) (manual outlining of the border, yellow) at the deep capillary plexus (DCP) was larger in the study eye (1.31 mm^2^) than in the fellow eye (0.93 mm^2^). (**E, F**) Vascular density at DCP was lower in the study eye (31.70%) than in the contralateral eye (34.19%).

Table 2 provides ocular characteristics of eyes in this study 3 months after silicone oil removal in comparison with the contralateral eyes. The mean logarithm of minimal angle of resolution (logMAR) best-corrected visual acuity (BCVA) was significantly lower in study eyes (0.51 ± 0.42) compared with fellow eyes (0.16 ± 0.19, *P* < 0.001). With regards to OCT parameters, the mean central foveal thickness (CFT) was significantly lower in study eyes (243.55 ± 36.76 μm) than those of the fellow eyes (265.06 ± 28.55 μm, *P* = 0.015). The mean macular ganglion cell-inner plexiform layer (mGCIPL) thickness was also significantly lower in study eyes (66.13 ± 16.54 μm) than those of the unaffected fellow eyes (78.13 ± 9.77 μm, *P* < 0.001). Among OCTA parameters, the mean foveal avascular zone (FAZ) area in the deep capillary plexus (DCP) was larger (0.73 ± 0.32 mm^2^ vs. 0.60 ± 0.22 mm^2^, *P* < 0.001) and the parafoveal mean vascular density (VD) in the DCP was lower (32.43 ± 4.24% vs. 34.43 ± 3.10%, *P* = 0.022) in study eyes than those of the fellow eyes. There was no significant difference in the mean FAZ area in the superficial capillary plexus (SCP) (*P* = 0.158) and the mean VD in the SCP (*P* = 0.873) between study eyes and fellow eyes. A representative case is shown in Fig. 1. Repeatability of the measurement between the two graders for the FAZ area was excellent for the SCP (intraclass correlation coefficient [ICC] = 0.948 in study eyes, ICC = 0.993 in fellow eyes, *P* < 0.001) and DCP (ICC = 0.928 in study eyes, ICC = 0.993 in fellow eyes, *P* < 0.001).

**Table 2:** Comparisons of retinal thickness and microvascular structure in study eyes treated with silicone oil and unaffected contralateral eyes.

	Study eyes	Contralateral eyes	*P*-value*
BCVA (logMAR)	0.51 ± 0.42	0.16 ± 0.19	**<0.001**
CFT (μm)	243.55 ± 36.76	265.06 ± 28.55	**0.015**
mGCIPL thickness (μm)	66.13 ± 16.54	78.13 ± 9.77	**<0.001**
SCP FAZ (mm^2^)	0.26 ± 0.11	0.23 ± 0.10	0.158
DCP FAZ (mm^2^)	0.73 ± 0.32	0.60 ± 0.22	**<0.001**
SCP VD (%)	34.12 ± 3.07	34.22 ± 2.60	0.873
DCP VD (%)	32.43 ± 4.24	34.43 ± 3.10	**0.022**

BCVA = best-corrected visual acuity; logMAR = logarithm of minimal angle of resolution; CFT = central foveal thickness; mGCIPL = macular ganglion cell-inner plexiform layer; SCP = superficial capillary plexus; FAZ = foveal avascular zone; DCP = deep capillary plexus; VD = vascular density.

Significant values with *P* < 0.05 are in bold. Values are expressed as mean ± SD unless otherwise indicated. *Paired *t*-test.

**Figure 2:**
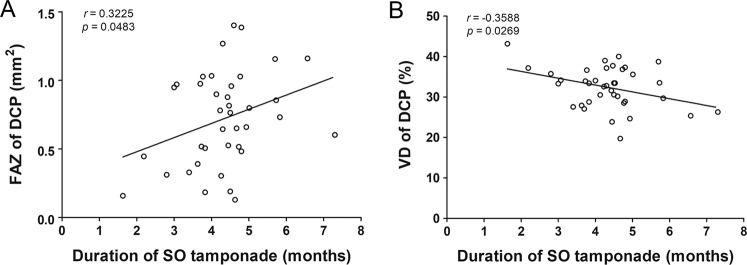
Scatter plots showing correlations between the duration of silicone oil (SO) tamponade and foveal avascular zone (FAZ) area of the deep capillary plexus (DCP) (**A**), and between the duration of SO tamponade and vascular density (VD) of the DCP (**B**). Pearson’s correlation coefficient (*r*) and *P* values for the slope of the regression line are noted.

Univariate and multivariate regression analysis were performed using the FAZ area of the DCP (Table 3) and VD of the DCP in study eyes (Table 4) as a dependent variable. The univariate regression analysis showed that duration of silicone oil tamponade (*P* = 0.048) and mGCIPL thickness (*P* = 0.095) affected the increase in FAZ area of the DCP. The multivariate regression analyses showed that only the duration of silicone oil tamponade (*P* = 0.034) was significantly correlated with the FAZ area of the DCP in the study eyes. Regarding the VD, the univariate regression analysis showed that volume of silicone oil injected (*P* = 0.140) and duration of silicone oil tamponade (*P* = 0.027) caused a decrease in VD of the DCP. The multivariate regression analyses showed that only the duration of silicone oil tamponade (*P* = 0.015) was significantly correlated with the VD of the DCP in study eyes. The duration of silicone oil tamponade was positively correlated with the FAZ area of the DCP (*r* = 0.3225; *P* = 0.0483), and it was negatively correlated with the VD of the DCP (*r* = −0.3588; *P* = 0.0269) (Fig. 2). FAZ area of the DCP (*P* = 0.381) and VD of the DCP (*P* = 0.528) did not correlate with LogMAR BCVA postoperatively (data not shown).

**Table 3:** Univariate and multivariate linear regression analysis for the foveal avascular zone area at deep capillary plexus.

Variable	β ± SE	*P* Value	β ± SE	*P* Value
	Univariate Analysis		Multivariate Analysis	
Age	0.003 ± 0.543	0.498		
Diabetes	−0.091 ± 0.740	0.514		
Hypertension	−0.016 ± 0.725	0.899		
Axial length	0.003 ± 0.796	0.942		
Duration of RRD before surgery	0.002 ± 0.739	0.668		
Preoperative factors				
Macula off	−0.094 ± 0.787	0.454		
Number of quadrants involved	−0.080 ± 0.939	0.370		
PVR	0.008 ± 0.723	0.952		
Intraoperative factors				
Combined cataract surgery	0.144 ± 0.645	0.200		
Perfluorocarbon liquid use	0.102 ± 0.632	0.544		
Operation time	0.000 ± 0.753	0.870		
Volume of SO injected	0.035 ± 0.501	0.675		
Postoperative factors				
IIOP during SO tamponade	−0.165 ± 0.782	0.155		
Duration of SO tamponade	0.103 ± 0.273	0.048	0.112 ± 0.051	**0.034**
CFT (μm)	0.000 ± 0.651	0.870		
mGCIPL thickness (μm)	0.005 ± 0.344	0.095	0.006 ± 0.003	0.089

RRD = rhegmatogenous retinal detachment; PVR = proliferative retinopathy; SO = silicone oil; IIOP = increased intraocular pressure; CFT = central foveal thickness; mGCIPL = macular ganglion cell-inner plexiform layer.

Significant values with *P* < 0.05 are in bold.

Overall *R*^2^ = 0.273, step-wise method.

**Table 4:** Univariate and multivariate linear regression analysis for the vascular density at deep capillary plexus.

Variable	β ± SE	*P* Value	β ± SE	*P* Value
	Univariate Analysis		Multivariate Analysis	
Age	−0.079 ± 0.065	0.234		
Diabetes	−0.506 ± 2.032	0.805		
Hypertension	−0.331 ± 1.882	0.861		
Axial length	0.174 ± 0.602	0.774		
Duration of RRD before surgery	0.042 ± 0.070	0.547		
Preoperative factors				
Macula off	−0.383 ± 1.827	0.835		
Number of quadrants involved	−0.832 ± 1.296	0.525		
PVR	−0.546 ± 1.948	0.781		
Intraoperative factors				
Combined cataract surgery	−0.265 ± 1.660	0.874		
Perfluorocarbon liquid use	−1.303 ± 2.443	0.597		
Operation time	−0.009 ± 0.023	0.707		
Volume of SO injected	1.765 ± 1.169	0.140	2.048 ± 1.094	0.070
Postoperative factors				
IIOP during SO tamponade	−2.043 ± 1.685	0.233		
Duration of SO tamponade	−1.686 ± 0.731	0.027	−1.820 ± 0.710	**0.015**
CFT (μm)	0.000 ± 0.026	0.994		
mGCIPL thickness (μm)	−0.013 ± 0.051	0.803		

RRD = rhegmatogenous retinal detachment; PVR = proliferative retinopathy; SO = silicone oil; IIOP = increased intraocular pressure; CFT = central foveal thickness; mGCIPL = macular ganglion cell-inner plexiform layer.

Significant values with *P* < 0.05 are in bold.

Overall *R*^2^ = 0.208, step-wise method.

## Discussion

Using OCTA, we provide evidence that silicone oil injection for the treatment of RRD could cause changes in the foveal microvascular structures, larger FAZ area and lower VD in the DCP. A multivariate regression analysis demonstrated that the duration of silicone oil tamponade is significantly correlated with enlargement of FAZ and reduction of VD in the DCP. Furthermore, the mGCIPL thickness and CFT were significantly thinner in study eyes than in the fellow eyes. To the best of our knowledge, this is the first study investigating the changes in foveal microvascular structures after RRD surgery with silicone oil injection using OCTA.

Our study showed that, 3 months after silicone oil removal surgery, the mGCIPL thickness and CFT were significantly thinner in study eyes than in the fellow eyes, which is consistent with previous studies. Christensen and la Cour^10^ demonstrated that thinning of the inner retinal thickness, which measures from the retinal nerve fiber layer to the outer plexiform layer, was observed in eyes operated with silicone oil compared to those with gas. Caramoy *et al*.^12^ also reported that mGCIPL contributes the most to inner retinal layers thinning in eyes that received silicone oil tamponade. More recently, Tode *et al*.^14^ revealed that thinning of the inner retinal layers, possibly evoked by silicone oil tamponade, might provide explanation for unexplained vision loss in these patients. Thus, our data confirmed previous findings that the mGCIPL thickness is significantly thinner in eyes operated with silicone oil injection for the treatment of RRD.

Notably, the mean FAZ area in the DCP was larger and the mean VD in the DCP was lower in eyes after silicone oil injection than those of the fellow eyes in current study. There have been several studies exploring the OCTA features in eyes with RRD. Sato *et al*.^15^ reported that there was a significant correlation between the FAZ area of the SCP and central retinal thickness in eyes treated with vitrectomy for macula-off RRD. However, this study did not analyze the FAZ area or VD of the eyes with RRD and did not show the specific endotamponade used in vitrectomy. Agarwal *et al*.^16^ investigated OCTA features in patients with macula-off RRD who underwent scleral buckling or vitrectomy with gas tamponade and compared them with healthy subjects. They revealed that mean capillary density index were reduced and FAZ area were enlarged in both the SCP and DCP in patients after surgery. In current study, we only included the patients with silicone oil as an endotamponade in RRD repair and therefore, could not be directly compared to the results published by Agarwal *et al*. However, enlargement of FAZ and reduction of VD in study eyes are consistent with the findings of the study by Agarwal. In our study, 71.1% of macula-off RRD patients were included, so we are aware that these findings have to be interpreted with caution because RRD itself in the absence of silicone oil endotamponade could induce the observed changes. In order to further investigate the impact of RRD itself, a pilot study was performed in six patents with unilateral RRD and had undergone vitrectomy with gas tamponade. The results showed that, compared to healthy contralateral eyes (61.66 ± 10.59 μm), eyes with gas tamponade exhibited no significant thinning of the mGCIPL thickness (56.67 ± 16.92 μm, *P* = 0.827). There were no significant differences in the mean FAZ area in DCP (0.89 ± 0.39 vs. 0.58 ± 0.24 mm^2^, *P* = 0.078) and the mean VD in the DCP (37.21 ± 6.89 vs. 38.52 ± 5.19%, *P* = 0.810) between eyes with gas tamponade and fellow eyes. These results suggest that changes of the foveal microvascular structures may be primarily attributed to the effect of silicone oil, not to the RRD itself, although these results are derived from a small sample size. Furthermore, it is reasonable to speculate that changes in foveal microvascular structures may be attributable to the effect of silicone oil, because duration of silicone oil tamponade is independently correlated with these changes.

Although the exact mechanism that causes enlargement of FAZ, reduction of VD in the DCP and mGCIPL thinning in eyes receiving silicone oil due to RRD was not determined, several causes might be involved in this phenomenon. Firstly, retinal ganglion cell damage could be induced by silicone oil. Previous studies have suggested a harmful effect of silicone oil to retinal structures, and toxic substances in removed silicone oil have been described^17,18^. It has been demonstrated that silicone oil damages the retinal tissue by mechanical stress to the fovea during prone position^19^. In addition, decreased VD might also reflect neuronal damage induced by the mechanical insult of silicone oil. Secondly, retinal thinning and vascular insufficiency might be attributable to photo-toxicity by foveal light exposure. Dogramaci *et al*.^20^ investigated the laboratory model and suggested that macular light exposure is further increased at the time of removal of silicone oil under direct microscope light. They revealed that eyes with silicone oil are particularly vulnerable to transient increase in light exposure because higher-energy light is transmitted through silicone oil and becomes potentially incident on already-stressed photoreceptors. García-Ayuso *et al*.^21^ investigated the damage produced by light in albino retinas and showed that significant death of retinal ganglion cells was caused by axonal strangulation by displaced retinal vessels. Thirdly, idiopathic reactions to silicone oil or retinal ionic environmental changes could contribute to retinal thinning and vascular insufficiency. Mechanism for toxicity proposed is potassium accumulation which can be due to the failure of potassium siphoning by Müller cells out of retina^22^. Additionally, Asaria *et al*.^23^ demonstrated that the toxic effect of silicone oil could be a result of pro-inflammatory cytokines accumulation in the retro-silicone oil fluid. Diffusion of metabolites and water-soluble cytokines away from the retina is decreased in silicone oil-filled eye^22,23^. These could have a detrimental effect on retinal ganglion cell or foveal microvascular structures at the time of tamponade, or during sudden re-equilibration at the time of silicone oil removal.

Interestingly, enlargement of FAZ and reduction of VD were more prominent in the DCP than in the SCP, potentially accounting for the retinal capillary network characteristics. The DCP resides within the so-called watershed zone where the oxygen level is significantly lower than that in the inner and outer retinal layer and might be more susceptible to ischemia^24,25^. Gonvers *et al*.^19^ investigated the effect of liquid silicone on rabbit retina and found that the most dramatic changes occurred around the outer plexiform layer, where the DCP is embedded. Accordingly, these observations suggest that the altered architecture of outer plexiform layer induced by silicone oil could be related to vascular insufficiency of the DCP in this study. Unfortunately, FAZ area and VD of the DCP were not correlated with visual outcomes. Taking into account that postoperative visual outcomes in RRD eyes following surgery might be influenced by various factors, including the macular involvement, duration of retinal detachment, or foveal photoreceptor integrity, it is plausible that this result was attenuated by other factors. It is also important to recognize that the duration of silicone oil tamponade is strongly correlated with the enlargement of FAZ and the reduction of VD in the DCP. These results suggest that the removal of silicone oil, performed as early as possible, could help avoid vascular insufficiency in the DCP.

There are several limitations to our study. First, this study was retrospective and a selection bias could have accentuated some estimates and masked others. Second, the sample size was small and the follow-up periods were short. Further studies with increased patient numbers and a longer follow-up duration will be needed to confirm our results. Third, it remains unclear whether the relationship between retinal thinning and vascular insufficiency is causal or coincidental. Finally, the possibility that current OCTA findings are due to RRD itself cannot be excluded, as current study included eyes with macula-off RRD as well as macula-on RRD. Future prospective studies with larger sample size adequate enough to conduct subgroup analyses by macular involvement and tamponading agents could provide more insight into the precise mechanism by which silicone oil may be related to foveal microvasculature and retinal thinning.

In conclusion, OCTA analyses following vitrectomy in eyes with RRD receiving silicone oil demonstrated marked changes of the foveal microvascular structures compared with normal contralateral eyes. The duration of silicone oil tamponade significantly correlated with the enlargement of FAZ and the reduction of VD in the DCP. Further studies are warranted in the future.

## Methods

### Participants

This retrospective study was performed at the Jeju National University Hospital in Korea. The study followed by tenets of the Declaration of Helsinki and was approved by the Institutional Review Board at Jeju National University Hospital (IRB approval number: 2017-11-002). The institutional review board waived informed consent due to the retrospective study design. We reviewed the medical records of patients with unilateral RRD who underwent surgical repair in our institution between March 2011 and December 2017. Patients were included if they underwent successful vitrectomy with silicone oil tamponade for a unilateral RRD and were followed for ≥3 months after silicone oil removal. Subjects were excluded if any of the following were present: (1) coexisting ocular condition that could potentially impair visual function (e.g., diabetic retinopathy/uveitis with macular edema, macular hole, comorbid maculopathy); (2) history of ocular trauma; (3) high myopia (spherical equivalent of ≥−6.0 diopters or axial length ≥ 26 mm); (4) glaucoma; (5) PVR grade greater than C;^26^ (6) bilateral RRD; (7) anisometropia > 2.0 diopters; (8) development of silicone oil emulsification; (9) development of ERM or macular edema; (10) second surgery due to failure of retinal reattachment; (11) optical media opacity that could significantly interfere with OCT image acquisition.

### Ocular examination

All patients underwent comprehensive perioperative ophthalmic examinations including measurement of BCVA, IOP with Goldmann applanation tonometry, slit-lamp biomicroscopy, indirect fundus examination, ultra-wide-field fundus photography (Optos 200Tx; Optos PLC, Scotland, UK), SD-OCT (Cirrus 4000; Carl Zeiss Meditec, Dublin, CA), and swept-source OCTA (PLEX Elite 9000; Carl Zeiss Meditec, Dublin, CA). The BCVA measurements were converted to logMAR units before analysis.

### Image analysis

The SD-OCT images were obtained and CFT was measured by macular cube 512 × 128 scanning protocol at 250 μm intervals in the center 4 mm to reconstruct a surface map of the 9 Early Treatment Diabetic Retinopathy Study (ETDRS) region^27^. The built-in ganglion cell analysis (GCA) algorithm was used to obtain the average mGCIPL thickness at center of the fovea. Only good-quality images with a signal strength of at least 7 and without segmentation failure or blinking artifacts were included in the analysis.

The OCTA images were obtained using swept-source OCTA. The FAZ area and parafoveal VD of the SCP and DCP were used to represent foveal microvascular structures. To measure the FAZ area and VD, a scan area of 3 × 3 mm (a 320 × 320 pixel array) was chosen at the center on the fovea. Automated segmentation of the full thickness retinal layer into the SCP, DCP, outer vascular retina, and choriocapillary vascular layers was performed by built-in software program to generate en face projection images. The FAZ area and VD were analyzed using Image J software (National Institutes of Health, Bethesda, MD) in order to binarize the OCTA images^28,29^. The FAZ area was measured by manual delineation and were calculated as [(pixels of FAZ) × (3/320)^2^] in mm^2^ ^30,31^. VD was defined as the percentage of the area occupied by vessels in binarized images. In the en face OCTA images, the average size of 20 pixels^2^ (equal to 0.0002 mm^2^) was set as a threshold indicating the area of no flow^32^. Subsequently, the images were automatically adjusted to threshold using the Niblack method in Image J. Each 320 × 320 pixels, 8-bit image was binarized to calculate the percentage of black and white pixels. Using the “Analyze Particles” tool, VD was calculated as the percentage of the portion of white pixels against the whole scan area^33,34^. All SD-OCT and OCTA images were evaluated by masked graders (J.Y.L. and E.K.L.) independently. Using SD-OCT and en face OCTA images, postoperative CFT, mGCIPL thickness, FAZ area and VD were measured 3 months after silicone oil removal and were compared with those of the unaffected contralateral eyes.

### Intraoperative procedures

Pars plana vitrectomy and posterior hyaloid membrane peeling using a 25-gauge vitrectomy system were performed by two experienced retinal surgeons (J.Y.K. and E.K.L.). After fluid-air exchange, endolaser photocoagulation was performed, and silicone oil was injected into the vitreous cavity. The same brand of silicone oil was used in all cases (Oxane 1300; Bausch & Lomb, Inc., NY). Patients were advised to maintain in the face-down position for two weeks postoperatively. After at least 3 months of silicone oil tamponade, silicone oil was removed by the pars plana approach after retinal attachment has been confirmed in the eyes.

### Statistical analysis

Statistical analysis was performed using the SPSS software (version 20; SPSS, Inc., Chicago, IL, USA). A paired t-test was used to compare SD-OCT and OCTA parameters between the treated and the contralateral eyes. To identify the factors related to FAZ area and VD of the DCP in study eyes, the potential determinants were tested using univariate and multivariate linear regression analyses. Variables with a significance of *P* < 0.15 in the univariate analysis were entered into the multivariate analysis. Correlations were analyzed using the Pearson test. The ICC was used to determine the intergrader reproducibility for the manually measured FAZ area. A 95% confidence interval (CI) and 5% level of significance were adopted. A *P* value less than 0.05 was considered statistically significant.

## Data Availability

Data supporting the findings of the current study are available from the corresponding author on reasonable request.
